# Decoding target discriminability and time pressure using eye and head movement features in a foraging search task

**DOI:** 10.1186/s41235-025-00657-y

**Published:** 2025-08-22

**Authors:** Anthony J. Ries, Chloe Callahan-Flintoft, Anna Madison, Louis Dankovich, Jonathan Touryan

**Affiliations:** 1 Humans in Complex Systems, U.S. Army DEVCOM Army Research Laboratory, 7101 Mulberry Point Rd, Aberdeen Proving Ground, MD 21005 USA; 2 Warfighter Effectiveness Research Center, U.S. Air Force Academy, Colorado Springs, CO 80840 USA

**Keywords:** Virtual reality, Eye tracking, Head movements, Visual search, Task prediction, Cognitive state, Visual foraging, Multiclass classification, Machine learning, Support vector machine

## Abstract

In military operations, rapid and accurate decision-making is crucial, especially in visually complex and high-pressure environments. This study investigates how eye and head movement metrics can infer changes in search behavior during a naturalistic shooting scenario in virtual reality (VR). Thirty-one participants performed a foraging search task using a head-mounted display (HMD) with integrated eye tracking. Participants searched for targets among distractors under varying levels of target discriminability (easy vs. hard) and time pressure (low vs. high). As expected, behavioral results indicated that increased discrimination difficulty and greater time pressure negatively impacted performance, leading to slower response times and reduced d-prime. Support vector classifiers assigned a search condition, discriminability and time pressure, to each trial based on eye and head movement features. Combined eye and head features produced the most accurate classification model for capturing tasked-induced changes in search behavior, with the combined model outperforming those based on eye or head features alone. While eye features demonstrated strong predictive power, the inclusion of head features significantly enhanced model performance. Across the ensemble of eye metrics, fixation-related features were the most robust for classifying target discriminability, while saccadic-related features played a similar role for time pressure. In contrast, models constrained to head metrics emphasized global movement (amplitude, velocity) for classifying discriminability but shifted toward kinematic intensity (acceleration, jerk) in time pressure condition. Together these results speak to the complementary role of eye and head movements in understanding search behavior under changing task parameters.

**Supplementary Information:**

The online version contains supplementary material available at 10.1186/s41235-025-00657-y.

## Introduction

In modern military operations, making quick, accurate decisions in visually complex environments is critical to mission success. This is particularly true for soldiers in dynamic, high-stress scenarios, requiring rapid ability to locate targets (e.g., potential threats in the environment) and differentiate them from distractors, which are sometimes visually similar (e.g., friendlies among enemy soldiers). The significant consequences of delayed or incorrect responses in these situations underscore the critical role of a soldier’s attentional focus and their capacity to manage competing, time-sensitive demands. Collectively, these factors shape how soldiers perceive and respond to information, ultimately affecting their decision-making and the effectiveness of their actions.

The military has shown an increasing interest in leveraging the computational power of artificial intelligence (AI) to help soldiers in complex, time-constrained decision-making. However, seamless integration with human teams requires providing AI with ongoing contextual information, such as changes in mission parameters or operator goals. This contextual information can potentially be inferred from changes in observable behaviors and human–system interactions. For example, delays in operator response times or atypical information requests from the AI may indicate that task parameters or environmental conditions have changed. Having this information and drawing such inferences would enable the AI to provide tailored assistance without imposing additional cognitive load on the operator by requiring explicit reports of task parameter changes. Real-time measurement and prediction of task-induced changes in search performance could significantly enhance decision-making, enabling effective adaptation to situational demands (Brunyé et al., 2020). For instance, AI could identify imbalances in cognitive load across a team and recommend task reallocation. As the military integrates AI into combat scenarios, detecting fluctuations in search behavior could inform autonomous systems of changes in the soldier’s cognitive state or mission parameters, allowing the AI to adjust its behavior for real-time support to improve team efficiency (Marathe et al., 2018; Metcalfe et al., 2021; Reis et al., 2021).

Changes in search behavior often reflect underlying shifts in cognitive state (Boot et al., 2006; Doshi & Trivedi, 2012; Eckstein, 2011; HEnderson et al., 2013; MacInnes et al., 2018). These changes can originate from both endogenous factors such as fatigue and memory (Ganesan et al., 2018; Le-Hoa Võ & Wolfe, 2015) and exogenous factors of the search array itself, like visual saliency (Christ & Abrams, 2006; Itti & Koch, 2000; Theeuwes, 1994; Yantis & Jonides, 1990). Classically, search behavior differences due to changes in task parameters have been studied using central tendency metrics calculated from a block of trials (e.g., average response times are slower when targets are heterogeneous compared to homogeneous, (Duncan & Humphreys, 1989). While this method is effective and has provided insight into numerous cognitive mechanisms, it does not allow for a trial-by-trial assessment of task-induced behavioral variance. Understanding these task-induced changes in behavior are essential to infer or “predict” the current task parameters. It is this prediction that could have meaningful impact in the design of future AI integration into human teams. As such, the current work seeks to demonstrate whether head and eye movement data (either together or separately) can be used to infer the current parameters of the human’s search task.

### Eye and head movements

Human eye gaze provides numerous metrics for assessing changes in search performance and information processing demands (GilChrist & Harvey, 2006; König et al., 2016; Marshall, 2007). Saccade characteristics, such as frequency and peak velocity, predict target detection accuracy and reflect workload variations during visually complex decision-making tasks (Boot et al., 2006; Di Stasi et al., 2011). Fixation metrics, including duration, frequency, and sequence, are strong predictors of target selection in object search tasks (Huang et al., 2015) and are effective for distinguishing between classes of images (Karessli et al., 2017). Scanpath length captures search strategies, with longer scanpaths suggesting a global or ambient processing approach that prioritizes information outside the immediate field of view (Groner et al., 1984; Velichkovsky et al., 2002). Lower decision certainty is associated with fewer, longer fixations and slower saccades (Brunyé & Gardony, 2017). In high-stress shooting scenarios, elite police officers exhibit longer fixations and faster response times on relevant targets compared to novices, reflecting more efficient search strategies (Vickers & Lewinski, 2012). Additionally, fixation frequency, duration, and pupil diameter reliably track fluctuations in cognitive workload (Enders et al., 2021; Mallick et al., 2016; Pomplun & Sunkara, 2019; Van Orden et al., 2001). Collectively, these gaze-based metrics illustrate how both spatial and temporal characteristics of eye movements can be used to evaluate task-induced changes in search behavior across various scenarios.

Head movements, though less frequently measured in visual search studies, complement eye-tracking metrics by providing unique information on the spatiotemporal characteristics of search behavior in visually complex and dynamic tasks (Agtzidis et al., 2019; Bischof et al., 2024; Callahan-Flintoft et al., 2021; Doshi & Trivedi, 2012). Head movements reveal broader shifts in attention and spatial orientation, especially during large gaze shifts or rapid reorienting (Pelz et al., 2001; Stahl, 2001b). Day to day visual search often requires scanning large regions of the visual field, using peripheral vision to detect and respond to multiple, potentially hidden targets. Integrating head movement metrics into search performance assessments, therefore, may enhance predictive power, contributing to more comprehensive and ecologically valid models of perception and decision-making.

Visual search in the real world occurs, most often, in a cluttered and dynamic three-dimensional environment where the head and eyes must move in concert to acquire necessary information for target detection. It is imperative then to explore these two movement systems in tandem for a deeper understanding of the cognitive processes underlying search behavior (Bischof et al., 2024; Sidenmark & Gellersen, 2019). Head and eye movements may differentially support cognitive function depending on whether task-relevant information lies in the peripheral or central regions of the current reference frame (David et al., 2020; Draschkow et al., 2021; Solman & Kingstone, 2014). For instance, when relevant information is spatially dispersed, participants may engage a trade-off between making head movements and engaging working memory storage (Draschkow et al., 2021). Additionally, individuals differ in their reliance on head versus eye movements during visual search, with some favoring head movements for reorienting; a distinction that may reflect different cognitive strategies, such as"head movers"versus"non-movers"(Fuller, 1992; Stahl, 2001a, 2001b). Together, these findings speak to the importance of both eye and head movements in supporting visual search execution and suggest that both systems may be affected by changes in task parameters.

### Virtual reality (VR) and visual foraging

Virtual reality (VR) offers unique capabilities to study visual search and other tasks that closely mimic real-world interactions (Bischof et al., 2024; Callahan-Flintoft et al., 2021; Clay et al., 2019). Traditional visual search paradigms, typically confined to two-dimensional displays, offer precise control over experimental variables but lack the immersive, spatial interactions essential for capturing natural head and eye movements. VR provides realistic, three-dimensional environments that replicate the spatial complexity of real-world search tasks, enabling the study of cognitive processes and the natural coordination of eye and head movements in controlled, yet realistic settings (Callahan-Flintoft et al., 2021, 2024; Clay et al., 2019; Sidenmark & Gellersen, 2019). Capturing both eye and head movements through integrated head-mounted display (HMD) systems, VR provides data on focal and peripheral search behavior which are essential elements of situational awareness in military operations (Brunyé & Giles, 2023; Pettersson et al., 2024; Sidenmark & Gellersen, 2019).

Similarly, visual foraging tasks provide an ecologically valid model for studying decision-making and attention allocation in multi-target scenarios (Cain et al., 2012; Kristjánsson et al., 2014; Wolfe, 2013). Unlike traditional single-target search tasks, visual foraging involves searching for and selecting multiple targets within a scene, reflecting real-world situations where multiple potential targets may arise. This complexity closely resembles challenges faced by soldiers who must identify and respond to multiple threats in combat, where prioritizing targets and filtering distractions is critical (Kristjánsson et al., 2022). Research shows that participants adapt their search strategies in foraging tasks based on target/non-target discriminability and time constraints, with more rapid switching between targets under high time pressure and less target switching when target discrimination is difficult (T. Kristjánsson et al., 2018). Such conditions are also associated with increased saccade amplitude and higher fixation frequency (Tagu & Kristjánsson, 2022). These findings highlight the flexibility of search strategies in foraging tasks and underscore the value of VR for examining cognitive processes underlying naturalistic visual search, particularly in environments where target discrimination and time pressure impose distinct task demands.

### Current study

The current study explores the potential of eye and head movement metrics to predict changes in target discriminability and time pressure. Participants engaged in a naturalistic foraging search task within a VR forest environment using a head-mounted display (HMD) equipped with integrated eye and head tracking. Participants were required to identify and engage targets among distractors under varying levels of discrimination difficulty (easy vs. hard) and time pressure (low vs. high). By analyzing the collected eye and head movement data, we aimed to determine the predictive power of these metrics in classifying task parameter changes.

Here, participants performed a foraging task while standing in one spot (i.e., without ambulation), enabling us to capture the natural coordination of eye and head movements during immersive search while still providing strict control on the spatial relationship with the viewer and the search array. This set-up serves as a midway point for ultimately understanding search in a fully ambulatory context. However, even though the participant is stationary, allowing the eyes and head to move freely during search provides a wealth of spatiotemporal features, any number of which could be influenced by changes in task parameters. As such, the current work adopts a data-driven approach in employing machine learning to disentangle whether eye and head movement is predictive of changes in search context. Support vector machines (SVM) and neural networks have been effective in classifying task-induced changes in behavior based on eye movement data (El Iskandarani et al., 2024; Hulle et al., 2024; Kothari et al., 2020; MacInnes et al., 2018). However, incorporating head movement metrics has the potential to significantly improve classification accuracy or provide an alternative for predicting behavioral changes when eye-tracking measurements are not feasible or practical (Hu et al., 2023). Recent evidence demonstrates that the temporal derivatives associated with head movements, velocity, acceleration, and jerk can predict task-relevant physiological responses in VR environments (Salehi et al., 2024). In addition to eye metrics, integrating head kinematic data, including rotation velocity, acceleration, jerk, and more traditional features such as amplitude, duration, and frequency, into machine learning models may capture complimentary information relevant to classifying search behaviors and provide insights that more classic analysis approaches could miss.

## Method

### Participants

The Institutional Review Boards at the U.S. Air Force Academy (USAFA) and the U.S. Army Combat Capabilities Development Command (DEVCOM) approved this study (ARL 21–132), and all procedures followed the guidelines of the Declaration of Helsinki. A total of 33 United States Air Force Academy (USAFA) cadets participated in the study. They were recruited through the Sona Systems subject pool and received course credit for participation. Two participants experienced technical difficulties with the system, and one experienced vertigo and did not complete the task. The data from these three participants were not analyzed. Of the 30 final participants, 24 were male, and 6 were female, average age of 19.1 years. Self-reported handedness showed 3 were left-handed, 26 were right-handed and 1 ambidextrous. Before beginning the experiment, participants provided written informed consent. To ensure participants had normal color vision they completed the Snellen chart (at least 20/40 vision required) and Ishihara color plates. Each of the 30 participants completed the vision test successfully without eyeglasses.

### Materials

The experiment was programmed in Unity (2020.3.44f1) and presented in the HTC Vive Pro Eye VR headset (1440 × 1600 pixels per eye, 90 Hz refresh, 110 deg field of view, VIVE SRanipal SDK) with embedded Tobii eye tracking (120 Hz sampling rate, Tobii XR SDK). The experimental environment was presented using a Corsair One PC (Windows 10, Intel Core i9 CPU @ 3.6 GHz, 64-bit, Nvidia GeForce RTX2080Ti, 32 GB RAM). Two external lighthouses tracked the head, the torso using a Vive Tracker, and hand controller positioned in a ForceTube gunstock (ProTube VR) to simulate a weapon.

### Design and procedure

After signing informed consent and undergoing the vision tests, participants completed a demographics form and the Simulator Sickness Questionnaire (SSQ) (Kennedy et al., 1993). A Vive tracker was then attached to the center of the chest, and the participant received general instruction on how to hold the weapon and perform the foraging task. Next participants were centered on a foam pad (2 × 3 ft) between the lighthouses. Participants could rotate their torso but were instructed to always keep their feet on the foam pad. The pad was used as it provided a proprioceptive reminder (i.e., the contrast between the foam and the floor) to participants to stay within the experimental space. Once positioned, the participant was handed the weapon controller to use in whatever hand they preferred and then the VR headset was donned and adjusted for fit and comfort. Next, participants adjusted the inter-pupil distance to ideal parameters determined by Tobii software and performed a 5-point eye-tracking calibration. After a successful calibration (determined by Tobii software), participants were given practice with the task to get familiar with the environment, the target and distractor objects, and aiming and shooting the weapon.

**Fig. 1:**
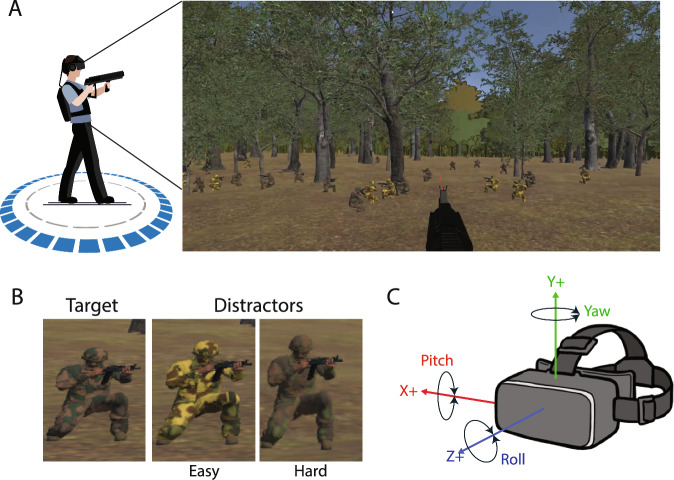
Task environment. **A**. Screenshot of the easy condition in the foraging shooting task. The red ray extending from the weapon indicates current aiming position. **B**. Examples of the search target and distractors in the easy and hard conditions. C. Spatial coordinates of the VR environment and head position (+ X right, + Y up, and + Z forward, left-handed coordinate system) with corresponding yaw, pitch, and roll rotations. Eye position data were recorded in a right-handed coordinate system (+ X left, + Y up, and + Z forward)

Participants performed a foraging search task within a forested environment (Meadow Environment—Dynamic Nature Version 2.9.3 created by Nature Manufacture) by shooting targets while trying to not shoot distractors. Participants shot the weapon by depressing the trigger on the hand controller, which produced an auditory gunshot. A visual ray (red line) extended from the weapon into the environment to assist aiming. Targets were kneeling avatars holding a weapon and wearing a specific camouflage pattern (Fig. 1). Targets were intermixed with distractors. Distractors were avatars also holding a weapon, in the same kneeling positions as targets, but wearing a camouflage pattern that was either easy to discriminate (Easy condition) or difficult to discriminate (Hard condition) from the target camouflage pattern. Four dominant colors were used in each avatar.^1^ Targets and distractors shot their weapons to generate background gunshot sounds but did not aim at the participant or fire projectiles in the virtual environment. If a target or distractor was shot, it fell to the ground and ceased shooting. The environment was rendered 360 degrees; however, camouflage targets and distractors were spawned without environment occlusion, only within 180 degrees of the game origin point (i.e., forward center position). Spawn points were a random distance between 10 (4.24 × 1.72 degrees of visual angle (dva)) and 100 m (1.1 × .5 dva). Trials were completed with low time pressure (45 s in the Low condition) or high time pressure (18 s in the High condition). Hard discrimination difficulty and high time pressure levels were determined a priori through pilot testing to ensure they were challenging yet accomplishable. The angular separation between targets within the scene varied by trial with half of the trials having a target dispersion of 5 dva and the other half 10 dva. For the purposes of this study, trials were collapsed across dispersion levels, leaving a 2 × 2 within-subjects design with target discriminability (Easy vs. Hard) and time pressure (Low vs Hard) as the main factors.

Each participant completed 4 blocks of 20 trials. Due to a pre-configuration error, one participant completed 6 additional trials having a target dispersion of 8 dva. These trials only occurred on the first block and were not included in the analysis. Each trial contained between 12 and 18 targets (uniformly distributed) and 12 distractors. This was implemented to prevent participants counting a fixed number of targets. Prior to each block a 5-point eye tracker calibration was performed. Each trial started with a blank background containing a plus sign + at the world forward position (0,0,1). When the gaze position remained continuously centered within 2dva and the head within 3dva of the central marker for 500 ms, the forested environment with targets and distractors was displayed. Participants were instructed to shoot all targets using the trigger button on the controller/weapon and press down on the controller track pad with their thumb when they were finished searching. If the trial time limit was reached (i.e., 45 or 18 s), the trial ended. Discriminability (as well as target dispersion) was randomized within each block with the constraint that each difficulty/dispersion trial combination was equally represented in the block. The first two blocks were low time pressure, and the last two blocks were high time pressure. Time pressure was blocked such that the first two blocks were always low pressure, and the final two blocks were high pressure. This fixed order was used to minimize carryover effects observed during pilot testing, where beginning with high pressure caused participants to adopt a sustained fast-paced search strategy that persisted into subsequent low-pressure blocks. Presenting low pressure first allowed participants to engage in more deliberate search behavior before transitioning into a more time-constrained strategy, better preserving the intended contrast between conditions. After the experiment the participants completed another SSQ as well as the three-component iGroup Presence Questionnaire (IPQ) (ScHubert, 2003). Summary results of the SSQ and IPQ can be found in Supplementary Materials.

### Data acquisition

Each unity frame, eye, head, and controller samples were timestamped, recorded, and synchronized with other experimental events (e.g., trial start/stop, target/distractor labels and positions) through the Lab Streaming Layer (LSL) and Lab Recorder (Kothe et al., 2024). LSL serves as a framework for synchronizing time-series data from various sources and sampling rates, offering a language-independent, real-time communication protocol for data recording and sharing. Due to a coding error, the Vive tracker used to track torso rotation did not record properly for any participant. Unfortunately, as a result, these data could not analyzed.

### Data processing

X, Y and Z coordinates from the left eye and head were used to calculate multiple metrics for the analyses (see Fig. 1 and Table 1) (Nyström & Holmqvist, 2010; Vlaskamp et al., 2005). Eye-tracking samples were deemed invalid if the pupil diameter was recorded as less than or equal to zero. If the time window of consecutive invalid samples was under 50 ms in duration, a linear interpolation was used to fill this gap. If the period extended longer than that, those samples, along with the neighboring 2 samples on either side of the drop-out, were marked as unusable and not included in saccade classification. Saccades and fixations were identified with a velocity-based algorithm adapted from the EYE-EEG toolbox (Dimigen et al., 2011; Engbert & Mergenthaler, 2006). Each participant’s eye unit vector was used to calculate angular velocity. The average velocity and standard deviation in each direction (x, y, z) were calculated, and a 3-dimensional, elliptical velocity threshold was set at six standard deviations from the mean in each direction. In this way, each participant had an automated, custom saccade threshold. Saccades were rejected if they were under 12 ms in duration (Hooge et al., 2007).

**Table 1:** Eye and head features used in classification models to infer task-induced effects of target discriminability and time pressure

Metric	Definition	Significance	Supporting Literature
**Fixation**			
Fixation Frequency	Total fixation count divided by trial time (sec)	**Fixation Frequency:** Reflects how often attention shifts to specific visual location. Higher frequencies may indicate increased visual samplingor search effort. **Fixation Duration:** Longer fixations typically reflect deeper processing, uncertainty, or hesitation; shorter durations may indicate more confident or automatic responses. **Fixation Duration Proportion:** Indicates how much of the trial is spent fixating; higher values suggest greter engagment or deliberation. **Target vs. Distractor Fixations:** Target-focused metrics reflect goal-directed attention while distractor-focused metrics may indicate inefficient search or difficulty in discrimination	Drew et al., (2017); Over et al., (2007); Reingold and Galhot, (2014); Lu and Sarter, (2019); Huanget al., (2015); Karessli et al., (2017); Iskandarani et al., (2024)
Avg Fixation Duration	Average fixation time (sec)		
Fixation Duration Proportion	Sum of all fixation durations divided by trial time		
Target Fixation Frequency	Total target fixation count divided by trial time		
Avg Target Fixation Duration	Average target fixation time (sec)		
Target Fixation Duration Proportion	Sum of all target fixation durations divided by trial time		
Distractor Fixation Frequency	Total distractor fixation count divided by trial time		
Avg Distractor Fixation Duration	Average distractor fixation time (sec)		
Distractor Fixation Duration Proportion	Sum of all distractor fixation durations divided by trial time		
Target Fixations per Target	Total number of target fixations divided by total number of targets		
Distractor Fixations per Distractor	Total number of distractor fixations divided by total number of distractors		
Target Distractor Fixation Ratio	Target fixation preference compared to other fixations		
**Saccade**			
Saccade Frequency	Total saccade count divided by trial time (sacades/s)	**Saccade Frequency:** Higher frequency may reflect rapid scanning or uncertainty in search strategy. **Saccade Size and Size per Second:** Larger or more frequent saccades may suggest a global search strategy while smaller saccades may indicate more focused, precise search behavior. **Target vs Distractor Saccade Size:** Differentiates the visual distance the eye travels totask-relevant vs. irrelevant stimuli, potentially reflecting discrimination efficiency	Watson et al., (2010); Over et al., (2007); Di Stasi et al., (2011); Velichkovski et al., (2002); Gordonet al., (2024)
Avg Saccade Size	Average size of a saccade (dva)		
Saccade Size Per Second	Sum of all saccade magnitudes divided by trial time (°/s)		
Avg Target Saccade Size	Average size of saccade to a target (dva)		
Avg Distractor Saccade Size	Average size of saccade to a distractor (dva)		
**Head**			
Head Movement Frequency	Total head movement count divided by trial time	**Head Movement Frequency and Size:** Capture how often and how far users reorient their field of view; higher values may relfect broader or more effortful search strategies. **Head Fixation Duration and Proportion:** Longer or more frequent head fixations may reflect more focused attention or reduced exploratory behavior. **Global Head Movement:** Indicates spatial engagement with the scene	Hu et al., (2023); David et al., (2018); Fuller, (1992); Stahl, (1999); Oommen and Stahl, (2005); Freedman and Sparks, (1997)
Avg Head Movement Size	Average size of a head movement (degrees of visual angle)		
Head Movement Size Per Second	Sum of all head movement magnitudes divided by trial time (°/s)		
Avg Head Fixation Duration	Average time the head remains below the threshold of a head movement (sec)		
Head Fixation Duration Proportion	Sum of all head fixation durations divided by trial time		
Head Fixation Frequency	Total head fixation count divided by trial time		
Global Head Movement Per Second	Sum of the angular distance the head moved divided by trial time Independent of head movement classification		
**Eye**			
Avg Eye in Head Angle	Average rotation of the eye within the head (dva)	**Eye in Head Angle:** Larger angles may reflect reliance on eye movements rather than head shifts. **Eye-to-Gaze Proportion:** important for undertanding search strategy preference (e.g."eye movers"vs."head movers")	Sidenmark & Gellersen, (2019, 2023); Stahl, (2001a, 2001b); Freedman, (2008); Zangemeister and Stark, (1982); Doshi and Trividi, (2012)
Global Eye Movement Per Second	Sum of the angular distance the eye moved divided by trial time. Independent of saccade classification		
Avg Eye to Gaze Proportion	Average proportion of eye contribution to gazeangle. Gaze Angle = Eye Angle + Head Angle		
**Kinematic**			
**Head**			
Avg Head Velocity	Average head velocity (°/s)	**Velocity:** Indicates speed of eye or head movement; higher values may reflect urgency or less deliberate behavior. **Acceleration:** Measures the rate of movement change. **Jerk:** Captures abruptnessor smoothness of movement; higher jerk suggests more reactive or less controlled movements. **Variability (Standard Deviation):** Grater variability across velocity, acceleration, or jerk may reflect less stable or more exploratory search strategies	Di Stasi et al., (2011); Salehi et al., (2024); Hu et al., (2023); Lisberger et al., (1981)
Std of Head Velocity	Standard deviation of head velocity (°/s)		
Avg Head Velocity X,Y,Z	Average head velocity around the X, Y, and Z axes (°/s)		
Std of Head Velocity X,Y,Z	Standard deviation of head velocity around the X, Y, and Z axes (°/s)		
Avg Head Acceleration	Average head acceleration (°/s^2^)		
Std of Head Acceleration	Standard deviation of head acceleration (°/s^2^)		
Avg Head Acceleration around the X,Y,Z	Average head acceleration around the X, Y, and Z axes (°/s^2^)		
Std of Head Acceleration around the X,Y,Z	Standard deviation of head acceleration around the X, Y, and Z axes (°/s^2^)		
Avg Head Jerk	Average head jerk (°/s^3^)		
Std of Head Jerk	Standard deviation of head jerk (°/s^3^)		
Avg Head Jerk X,Y,Z	Average head jerk around the X, Y, and Z axes (°/s^3^)		
Std of Head Jerk X,Y,Z	Standard deviation of head jerk around the X, Y, and Z axes (°/s^3^)		
**Eye**			
Avg Eye Velocity	Average eye velocity (°/s)		
Std of Eye Velocity	Standard deviation of eye velocity (°/s)		
Avg Eye Velocity X,Y,Z	Average eye velocity around the X, Y, and Z axes (°/s)		
Std Eye Velocity X,Y,Z	Standard deviation eye velocity around the X, Y, and Z axes (°/s)		
Avg Eye Acceleration	Average eye acceleration (°/s^2^)		
Std of Eye Acceleration	Standard deviation of eye acceleration (°/s^2^)		
Avg Eye Acceleration around the X,Y,Z	Average eye acceleration around the X, Y, and Z axes (°/s^2^)		
Std Eye Acceleration around the X,Y,Z	Standard deviation eye acceleration around the X, Y, and Z axes (°/s^2^)		
Avg Eye Jerk	Average eye jerk (°/s^3^)		
Std of Eye Jerk	Standard deviation of eye jerk (°/s^3^)		
Avg Eye Jerk X,Y,Z	Average eye jerk around the X, Y, and Z axes (°/s^3^)		
Std Eye Jerk X,Y,Z	Standard deviation eye jerk around the X, Y, and Z axes (°/s^3^)		

To exclude biologically implausible saccades, an additional set of thresholds was applied. Specifically, saccades longer than 120 ms or with peak velocities below 25 deg/sec or above 1000 deg/sec were removed (Enders et al., 2021; Holmqvist et al., 2011; Long et al., 2024). Saccades and head movements with amplitudes ≤ 1 deg and fixations (eye and head) shorter than 75 ms were also excluded from the analysis (Hooge et al., 2022). Saccade and fixation-based metrics were calculated after removing these outlier events.

To classify head movements, we applied an adapted algorithm from Chen and Walton (2005), originally designed to classify head movements in macaques but shown to be highly reliable when classifying human head movements (Callahan-Flintoft et al., 2024). Head movements were detected by applying a sliding 100 ms window to the head’s angular speed. Head motion onset began when at least 72% of data points within this window exceeded a threshold of 6°/s, with fewer than three consecutive time points falling below the threshold. Head movement onset was recorded as the first time point in the window exceeding the threshold. Similarly, motion offset was determined using a 22 ms window, where 72% of data points were below the threshold. The exact head movement offset time was defined as the first time point within this qualifying window that dropped below the threshold. Head ‘fixations’ were periods between classified head movements where the head sustained a low angular speed and remained relatively stable.

### Metrics calculation

Trial completion time, average inter-target engagement time and d-prime were evaluated to assess behavioral performance. Target completion time was measured from scene onset to when the participant pressed the trial end button indicating search was complete or when time expired, whichever came first. Inter-target time was the average time taken between successive target hits. D-prime was used to distinguish between signal and noise by quantifying the difference in hit and false alarm rates. This provided a better measure of sensitivity to the differences between targets and distractors while accounting for potential response bias when compared to target accuracy alone. Analyses were performed in R (RStudio 2023.12.1 + 402"Ocean Storm"Release).

Multiple metrics were computed using the rotational data of eye and head movements along the X (pitch), Y (yaw), and Z (roll) axes, with many derived across both effectors. We focused on core temporal and spatial characteristics of movement, including frequency, amplitude, and duration, features commonly used to characterize attentional engagement and search strategy in visually demanding tasks. For example, fixation duration and frequency have been linked to visual processing load and search efficiency (Drew et al., 2017; Reingold & Glaholt, 2014). Saccadic peak velocity has been proposed as an index for detecting variations in mental workload (Di Stasi et al., 2011), and saccade size has been shown to predict the onset time of object processing during visual search tasks (Gordon et al., 2024). Similarly, head movement amplitude and frequency reflect spatial reorientation and can signal increased search demands or target uncertainty (David et al., 2018; Freedman & Sparks, 1997; Oommen & Stahl, 2005). We also incorporated gaze and head kinematics which have shown promise in identifying cognitive state changes and user experience during complex VR tasks (El Iskandarani et al., 2024; Hu et al., 2023).

To capture the dynamics of eye and head movements more comprehensively, we calculated kinematic features based on velocity, acceleration, and jerk (Culmer et al., 2009; Di Stasi et al., 2011; Hu et al., 2023; Kothari et al., 2020; Lisberger et al., 1981; Plamondon, 1995; Salehi et al., 2024). Velocity refers to the rate of change of angular position over time. It provides insight into how quickly the eyes and head move during gaze shifts, reflecting the speed of orienting behavior. Velocity was computed as the first derivative of the angular position with respect to time for each axis. Acceleration is the rate of change of velocity over time. It indicates how rapidly the speed of eye and head movements increases or decreases, offering information about the smoothness and control of movements. Acceleration was calculated as the first derivative of velocity or the second derivative of angular position. Jerk represents the rate of change of acceleration over time. It captures the abruptness or smoothness of movements, with higher jerk values indicating more abrupt changes. Jerk is particularly relevant for detecting sudden adjustments in gaze direction. Jerk was computed as the first derivative of acceleration or the third derivative of angular position.

Eye-specific features were also considered, such as the average eye-in-head angle and the proportion of eye contribution to overall gaze, where gaze is defined as the sum of eye and head angles (Doshi & Trivedi, 2012; Freedman, 2008; Sidenmark & Gellersen, 2019; Sidenmark et al., 2023; Stahl, 1999, 2001b; Zangemeister & Stark, 1982). The proportion of eye contribution indicates the relative reliance on eye versus head movements during gaze shifts.

To glean insights into differences in information processing, separate metrics for target and distractor processing were also calculated, such as fixation frequency, duration on targets and distractors, and target-distractor fixation ratio. These metrics were exclusively available for eye measurements as head movements lack the same spatial granularity.

Where applicable, metrics were normalized by trial duration to account for differences in trial lengths. Each metric was calculated separately for each trial. See Table 1 for the complete list and descriptions of all computed metrics. Data files and processing scripts will be made available upon reasonable request.

### Machine learning classification

The Boruta algorithm, a multivariant wrapper feature selection method (Kursa et al., 2010), was used to assess feature importance prior to building classification models. Boruta identifies the most relevant features in a model-agnostic manner by comparing the importance of actual features to shuffled versions of the same data, referred to as"shadow features."A random forest model was run for 50 iterations, and the importance of each original feature was compared to the maximum importance of the shadow features in each iteration. At the end of the iterations, a binomial distribution (α = 0.05) was used to determine whether each feature was significantly important. By applying Boruta prior to running machine learning pipelines, we eliminated redundant and irrelevant features. Using only the important and independent features to train models reduces noise, helps avoid overfitting issues, and improves the human interpretability of model features (Dwivedi et al., 2024).

The subset of important features from Boruta were incorporated into a machine learning pipeline in Python using sklearn (Pedregosa et al., 2011) consisting of Standard Scaling and a support vector classifier (SVC) with a radial basis function kernel. Model validation was performed via a tenfold cross-validation method, which divides the data into 10 equal portions with roughly equivalent class distribution. In each fold, 9 of the portions are used to train the pipeline, and the last is used to test the model using classification accuracy as the primary evaluation metric.

To compare model performance, nonparametric Wilcoxon tests were conducted using the SciPy library (Virtanen et al., 2020). The Bonferroni–Holm modifier was applied to avoid false positives, and effect sizes were calculated using Hedges’ *g.*

While Boruta provides a binary indication of whether a feature is significant or not, it does not provide the importance ranking of these features on model predictions. Feature importance within the models was performed using Shapley additive explanations (SHAP) Python library (Scott & Su-In, 2017). This provided a ranking of the features selected through Boruta. As in prior research, we selected a 20% representative subset of samples to train SHAP (Christoph, 2020).

To investigate the contribution of both head and eye movements to model performance, as well as to tease apart how task parameter changes might affect movement systems differently, the following models were run. First, a 4-way classification omnibus model was run using all eye and head features designated as significantly important (Eye and Head). This model labeled each trial based on predicted target discriminability (Easy vs Hard) and time pressure (Low vs High), such that any given trial could be classified as Easy-Low, Easy-High, Hard-Low, or Hard-High. Next, a 4-way classification (with the same labels listed above) model was fit using only features derived from eye-tracking data (Eye Only) and another model was fit using only features derived from head-tracking data (Head Only). Finally, to assess whether eye and head features differ in their ability to infer changes in search parameters two separate 2-way classification models were run for each feature subset, focusing separately on target discriminability and time pressure.

## Results

### Eye and head movement summary

**Fig. 2:**
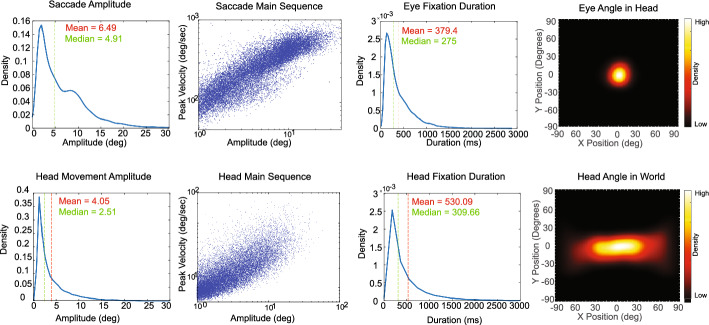
Eye (top row) and head movement (bottom row) summary from 20,000 randomly selected trial events across all participants. Left column—Saccade (top) and head movement (bottom) amplitude distribution. Second column from left—Main sequence plots for saccades (top) and head movements (bottom), illustrating the relationship between movement amplitude and peak velocity. Third column from left—distribution of eye and head fixation durations. Right column—heat map showing the angular distribution of eye in head (top) and head in the world (bottom). deg = degrees; ms = milliseconds

To demonstrate data quality, Fig. 2 provides a quantitative summary of eye and head movement characteristics. In the left column, the distribution of saccade and head movement amplitudes reveals a bimodal pattern for saccades, with average saccade amplitudes exceeding those of head movements. The second column illustrates main sequence plots for both eye and head movements, showing a clear velocity–amplitude relationship typical of these effectors (Bahill et al., 1975; Zangemeister et al., 1981). The fixation duration distributions display similar patterns for eye and head, though head fixations were generally longer on average than eye fixations. Angular distributions in the top right and bottom right plots show that eye position, relative to the head, remains within approximately 15 degrees of head center, while head orientation is predominantly distributed along the horizontal plane.

### Behavioral performance

**Fig. 3:**
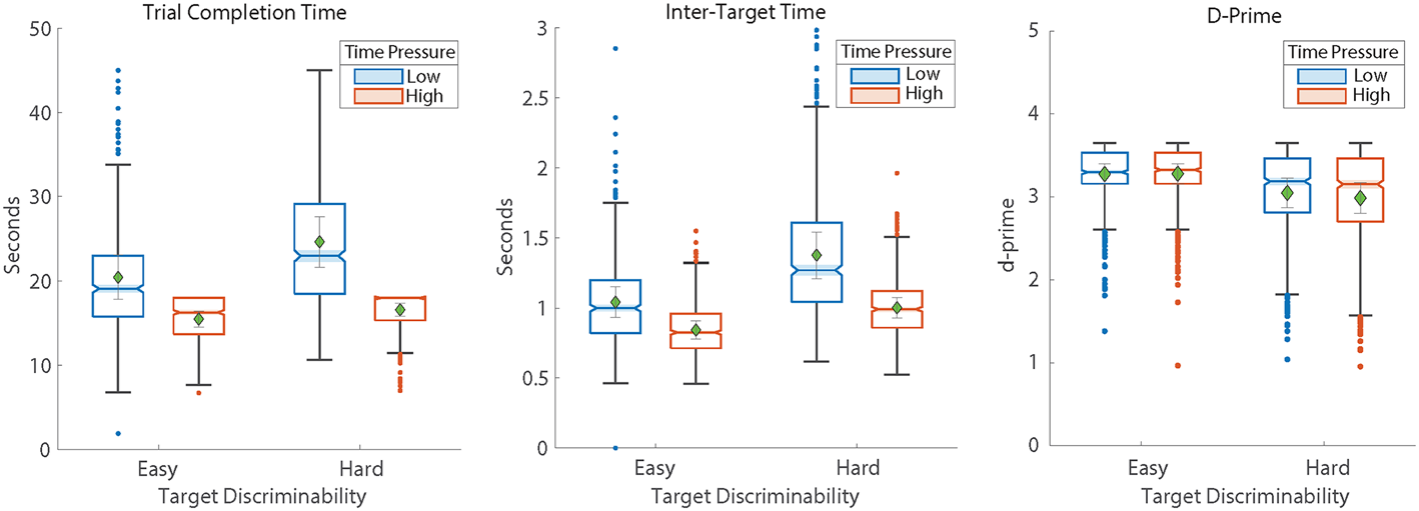
Behavioral performance. Target discriminability and time pressure had a significant main an interactive effect on trial completion time (left) and inter-target engagement time (middle). Target discriminability significantly impacted d-prime (right)

Dependent measures were analyzed using a random effects repeated measures analysis of variance (ANOVA) with the ‘ezANOVA'R package. The within-subjects factors were target discriminability (Easy, Hard) and time pressure (Low, High) with participant as a random effect (Fig. 3,^2^). Trial completion analysis revealed a significant main effect for target discriminability F(1,29) = 155.8, *p* <.001, η^2^_g_ =.11, with easy discrimination trials completed faster than hard. The main effect of time pressure was also significant F(1,29) = 65.2, *p* <.001, η^2^_g_ =.42 with high time pressure trials completed faster than low time pressure. The discrimination by time pressure interaction was significant F(1,29) = 96.5, *p* <.001, η^2^_g_ =.04 indicating an increased impact of time pressure in the difficult with respect to easy discrimination trials. Similar findings were found for inter-target time which showed main effects for target discriminability F(1,29) = 227.22, *p* <.001, η^2^_g_ =.33 and time pressure F(1,29) = 97.88, *p* <.001, η^2^_g_ =.39 indicating the average time between target engagement was faster for easy discrimination and high time pressure targets relative to hard discrimination and low time pressure targets. The interaction was significant F(1,29) = 40.9, *p* <.001, η^2^_g_ =.06 suggesting a larger effect of time pressure in the hard discrimination condition. The d-prime analysis revealed a main effect for target discriminability F(1,29) = 43.5, *p* <.001, η^2^_g_ =.17 showing higher d-prime in the easy compared to hard discrimination condition. However, the main effect for time pressure and the interaction between discrimination difficulty and time pressure were not significant (*p* >.05). Together these results show that target discriminability and time pressure significantly influenced behavioral performance, with faster completion and inter-target times and higher d-prime under easier conditions.

Behavioral data indicated that 38% of high time pressure trials reached the 18-s time limit, suggesting the trial ended automatically rather than by participant decision. However, this does not necessarily imply insufficient time to complete the task as many participants still achieved 100% hit rate, even at the maximum duration, indicating that they may have completed a first pass and continued scanning or verifying targets. Similarly, there was a wide range of hit rates in trials that ended earlier, suggesting that participants often had time remaining but chose to terminate. Notably, some of the lowest hit percentages occurred in trials participants ended on their own, further implying that time constraints were not the sole limiting factor. In contrast, only 3% of low time pressure trials ended due to the time limit, while 33% extended beyond 18 s, reinforcing the impact of time pressure on search behavior.

### Classification

**Fig. 4:**
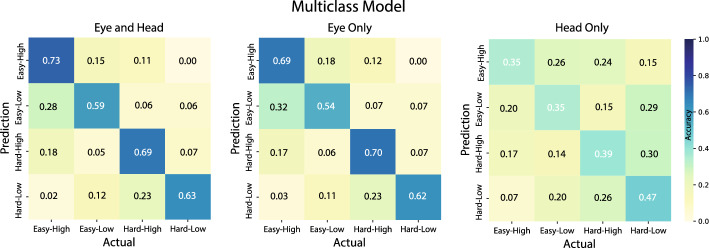
Multiclass model accuracy. Significant detection of task-induced effects of target discriminability and time pressure was achieved across all feature sets, with combined eye and head features having the highest model accuracy

The Boruta algorithm identified the most significant features from each feature set, and these were used in the 4-way classification (Multiclass model; Fig. 4). Model performance when using 49 of the 76 combined eye and head features was significantly better than random chance *(M* = 0.66, *SD* = 0.03, *p* = *.002 g* = *14.15).* Similarly, when using 39 out of 45 Eye Only features, the model achieved significant classification above chance (*M* = 0.64, *SD* = 0.03, *p* = *.002, g* = *7.70*) and 17 of 31 Head Only features, also performed significantly above chance in the 4-way classification model (*M* = 0.39, *SD* = 0.02, *p* = *.002*, *g* = *6.77*). Notably, incorporating head features with eye features significantly improved classification accuracy relative to Eye Only features (*p* = *0.03, g* = *0.93)* highlighting the modest but complementary value of combining head movement features with eye movement data. Eye Only features significantly outperformed Head Only features (*p* = 0.019, *g* = 5.61).

The top 10 features, ranked in order of importance, for each model are included in Table 2.

**Table 2:** Top 10 features and mean accuracy for each model

4-Way Classification				Target Discriminability		Time Pressure	
Feature Rank	Eye &Head M = 0.66	Eye M = 0.63	Head M = 0.39	Eye M = 0.85	Head M = 0.65	Eye M = 0.77	Head M = 0.64
**1**	Target Fixations per Target	Target Fixations per Target	Global Head Movement per Sec	Distractor Fixation Frequency	Global Head Movement per Sec	Target Fixations per Target	Avg Head Acceleration X
**2**	Distractor Fixations per Distractor	Distractor Fixations per Distractor	Std Head Velocity X	Distractor Fixation Frequency	Avg Head Velocity Z	Target Fixation Frequency	Std Head Velocity Z
**3**	Saccade Frequency	Saccade Frequency	Avg Head Velocity X	Distractor Fixations per Distractor	Avg Head Fixation Duration	Avg Target Fixation Duration	Std Head Velocity X
**4**	Distractor Fixation Duration Proportion	Distractor Fixation Duration Proportion	Avg Head Acceleration X	Target Fixations per Target	Avg Head Jerk	Saccade Size per Sec	Head Amplitude per Sec
**5**	Distractor Fixation Frequency	Target Fixation Frequency	Head Amplitude per Sec	Target Fixation Duration Proportion	Head Movement Frequency	Saccade Frequency	Std Head Jerk X
**6**	Target Fixation Frequency	Distractor Fixation Frequency	Head Fixation Frequency	Fixation Duration Proportion	Head Fixation Duration Proportion	Avg Eye Acceleration X	Avg head Fixation Duration
**7**	Fixation Duration Proportion	Target Fixation Duration Proportion	Avg Head Fixation Duration	Avg Eye Velocity X	Std Head Velocity X	Target Fixation Duration Proportion	Head Fixation Frequency
**8**	Target Fixation Duration Proportion	Fixation Duration Proportion	Avg Head Jerk	Avg Eye Jerk Y	Avg Head Velocity	Avg Eye Jerk X	Avg Head Jerk X
**9**	Avg Eye in Head Angle	Avg Eye in Head Angle	Avg Head Velocity X	Avg Eye Acceleration Y	Avg Head Velocity X	Distractor Fixations per Distractor	Avg Head Jerk
**10**	Saccade Size per Sec	Avg Eye Acceleration X	Avg Head Jerk X	Avg Saccade Size	Avg Head Movement Size	Avg Eye Velocity	Std Head Velocity Y

Avg = Average; Std = Standard Deviation; Sec = Second; See Table 1 for feature definitions

**Fig. 5:**
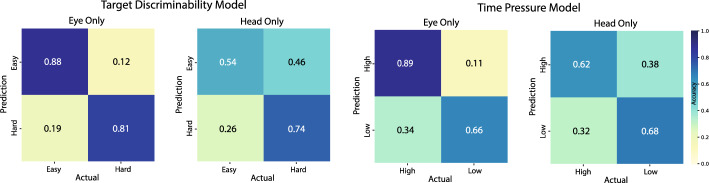
Classification accuracy for the target discriminability and time pressure models from Eye Only and Head Only features

Target discriminability and time pressure were modeled separately using either Eye Only or Head Only features, and all models performed significantly above chance (Fig. 5). The target discriminability model trained on Eye Only features achieved a mean accuracy of 0.85 (SD = 0.03, *p* = 0.002, g = 7.41), utilizing 34 of the 45 available eye features. In comparison, the time pressure model using Eye Only features reached a mean accuracy of 0.77 (SD = 0.02, *p* = 0.002, g = 8.57), with 35 of 45 features selected. Similarly, Head Only feature models also exceeded chance performance: The target discriminability model attained a mean accuracy of 0.64 (SD = 0.04, *p* = 0.001, g = 1.93) using 12 of the 31 available head features, while the time pressure model achieved 0.65 (SD = 0.04, *p* = 0.002, g = 2.48) with 20 of the 31 features. Eye movement features were better at classifying the target discriminability of a trial (*M* = 0.85, *SD* = 0.03) compared to the time pressure (*M* = 0.77, *SD* = 0.02), *p* = 0.002, *g* = 2.46. When using only head movement features, classification accuracies between target discriminability (M = 0.64, SD = 0.04) and time pressure (M = 0.65, SD = 0.04) were not statistically significant (*p* > 0.5).

## Discussion

The primary objective of this study was to investigate how task parameters—target discriminability and time pressure—affects foraging behavior and whether eye and head movement metrics can be used to reliably infer these task-induced changes. By employing a naturalistic VR foraging task, we aimed to examine the extent to which these movement systems contribute independently and collectively to adaptive search strategies under varying task conditions.

Behavioral results indicated that both target discriminability and time pressure significantly influenced search performance. Consistent with prior research, trials with easily discriminable targets from distractors (Easy condition) led to faster trial completion times, shorter inter-target times, and higher d-prime scores, indicating more efficient and selective search behavior (Kristjánsson et al., 2018; Tagu & Kristjánsson, 2022). In contrast, the hard condition was associated with slower response times and reduced d-prime, highlighting the greater perceptual demands required when targets were more visually similar to distractors. Time pressure also had a significant impact, with high-pressure trials showing faster completion and inter-target times particularly under harder discriminability. This interaction between difficulty and time pressure reflects the competing demands of speed and perceptual decision-making during visual search. Participants may shift toward a strategy that prioritizes speed over accuracy, suggesting a potential tradeoff between rapid decision-making and visual discrimination during search.

While it is well known that changes in task parameters, as manipulated here, produce changes in behavior, these metrics are typically reported as descriptive statistics in post hoc analyses, after averaging across multiple trials. To provide adaptive support, any intelligent system will need to use moment-to-moment human behavior to infer the given task and environmental parameters. As an intermediate step toward such a system, the current work used a machine learning approach to classify the target discriminability and time pressure level of individual trials. Our classification approach demonstrated that eye and head movement features can be used to accurately detect the task conditions that shape search behavior. As expected, eye features alone achieved high classification accuracy. However, when combined with head movement features, classification accuracy improved significantly. By identifying which metrics carry predictive value across trials, this work establishes a foundational feature space that could be translated into finer temporal windows in future work.

### The feasibility of using head tracking to infer task parameters

Modern eye-tracking devices, such as those in HMDs or mobile glasses, often include head-tracking capabilities, offering an additional resource for analyzing search behavior. By comparing Eye Only and Head Only models our findings suggest that head movement data alone can infer task-induced changes in search behavior, though with reduced accuracy compared to eye-tracking data. However, when combined with eye-tracking metrics, head movement data provided unique information, enhancing the overall predictive power of the model. This highlights the potential of head movement data as both a standalone substitute when eye tracking is unavailable and a complementary feature for enhancing the analyses of cognitive processes and search behavior in dynamic environments (Clay et al., 2019). Despite advances in camera-based mobile eye tracking, capturing relative or absolute gaze angle across varied real-world environments remains challenging due to factors like variable illumination or device slippage (Ghiani et al., 2024). In contrast, head-tracking sensors, such as accelerometers or IMUs, offer a more robust while less precise means to estimate gaze direction, making them an attractive alternative for capturing data in challenging environments. Our findings underscore the potential of head-tracking data to quantify task-induced changes in behavior, even when eye-tracking data are unavailable. Furthermore, the simplicity and durability of head-tracking sensors make them a practical solution for applications requiring lightweight, unobtrusive, and cost-effective systems.

The results from our multiclass classification showed head movement metrics provided unique information not fully captured by eye metrics alone. The most informative features in the 4-way classification model from head-only data were the total angular distance of head movement during the trial, normalized for trial duration, followed by kinematic features such as velocity and acceleration. Notably, head movement distance and stability (i.e., head fixation duration) were more sensitive to task-induced changes for target discriminability, while kinematic features were ranked higher for time pressure. These findings align with prior research demonstrating the utility of head kinematics for inferring search behavior (Hu et al., 2023; Salehi et al., 2024). For example, Hu et al. (2023) showed that head movement velocity and acceleration could reliably distinguish between different types of visual search, such as free viewing, target search, and tracking. Similarly, research by (Aivar et al., 2024), demonstrated that body movement velocity increases with repeated search in the same context, reflecting an optimization of movement strategies for faster and more efficient search execution. This, combined with the fact that there was no significant difference in performance between the 2-way classification models of target discriminability and time pressure using head data alone, suggests that head tracking may provide some, though limited insight into the parameters of an individual’s search.

### The role of eye movements in target discriminability and time pressure

In the target discriminability model, fixation behaviors emerged as the most informative features, including Distractor Fixation Frequency, Target Fixation Duration Proportion, and Distractor Fixation Duration Proportion. This pattern suggests that eye movement metrics are sensitive to the perceptual details between targets and distractors. Specifically, under more difficult conditions, participants likely increased feature-level inspection, fixating on distractors more frequently and for longer durations as they made their decision. This sensitivity indicates that eye movement behavior can serve as a dynamic, implicit signal of perceptual difficulty.

Conversely, the time pressure model prioritized fixation frequency and saccadic behaviors, with top features including Target Fixations per Target, Target Fixation Frequency, Saccade Size per Second, and Saccade Frequency. This suggests that under high time pressure, participants may have adopted a faster, more efficient search strategy with frequent saccades and brief fixations, likely to maximize the rate of information acquisition. Therefore, these eye movement features provide the potential for tracking cognitive state changes associated with search urgency.

The current results are consistent with previous findings that show eye movement metrics such as saccade amplitude, fixation duration, and gaze velocity are highly sensitive to changes in search demands and target discriminability. For example, increased saccade amplitude and fixation frequency have been linked to harder visual search tasks, where targets are more difficult to discriminate from distractors (Pomplun et al., 2013; Tagu & Kristjánsson, 2022). Similarly, fixation duration tends to increase as target discriminability becomes more challenging (Becker, 2011; Pomplun et al., 2013), while tasks with high perceptual and attentional demands decrease peak saccadic velocity (BacHurina & Arsalidou, 2022). Together our results are in agreement with current theories of visual search, such as the functional viewing field (FVF), which suggest that task demands, particularly search difficulty, modulate the amount of information processed during a fixation. These demands subsequently influence the spatial and temporal characteristics of saccades and fixations as participants adjust their strategies to effectively process visual information (Hulleman & Olivers, 2017; Young & Hulleman, 2013).

## Practical implications

The ability to infer task conditions from combined eye and head movement metrics has meaningful implications for the development of adaptive systems that monitor user state. IMUs, used to track head movements, are affordable, lightweight, and robust sensor. The current work indicates that such a sensor, easily deployed to the field, could provide insight into target discrimination difficulty experienced by a soldier triggering adjustments in system support. For example, this information could be shared with other squad members or a commander to inform the reallocation of resources such as deploying autonomous agents to aid in search and target interrogation. While head movement data provide valuable insight into user state, the results indicate that this signal alone is not as informative as eye movement data. Our results show that eye, compared to head data, provide higher classification accuracy and should be prioritized when available. In contexts with degraded or unavailable eye tracking, due to occlusion, motion, lighting, etc., head-tracking data offer a viable alternative.

Importantly, the utility of head movement data as a substitute will also depend on the search context. In constrained environments, such as narrow hallways, head movements are limited, and eye movements are more likely to carry the critical information needed for search. In contrast, in broader or more open environments where potential search targets fall beyond the customary oculomotor range (COMR), head movements become more informative and can better reflect search dynamics (Stahl, 1999, 2001b). Thus, inferring contextual information from head movement data is most appropriate when search demands extend beyond the natural range of eye movements alone.

## Limitations and future work

A notable aspect of our approach was the inclusion of target- and distractor-specific metrics, such as fixation frequency and duration, which emerged as some of the most discriminative features in our models. For example, Distractor Fixation Frequency was a top-ranked feature for target discriminability, reflecting participants’ increased reliance on distractor fixations when distinguishing targets in the hard condition. While the results remain meaningful and demonstrate that these features offer valuable insights into task-induced search behavior, environmental labels may not be readily available in real-world settings.^3^ However, our approach is a critical step toward developing a robust inferential model of task-induced changes in foraging behavior. Quantifying and understanding the utility of all eye and head movement metrics, regardless of their accessibility in a real-world context, provides a broad foundation for multimodal application spaces. For example, augmented reality (AR) applications would potentially have some context as to the task-relevance of overlaid information (e.g., a UI display) as well as some understanding of the environment (e.g., geospatial location). Head and eye movement features could be combined with other sensors, such as cameras, to provide a rich understanding of how environmental factors change behavior of individuals and groups. Moreover, having such a model to characterize “hard” or “easy” visual search may provide AI systems valuable information about the effectiveness of the current AI support. For instance, if eye and head movement signatures indicate an operator is having difficulty in searching the environment, this may indicate too much visual clutter in the AR display.

Several factors limit the direct applicability of our findings to more naturalistic scenarios. The current study was conducted in a stationary context, with the task confined to 180 degrees of space. While this setup allowed for controlled measurement of eye and head movements during the task, it may not fully capture the spatiotemporal demands encountered in all foraging search environments. Future work should examine the generalizability of these findings to more ambulatory tasks with natural navigation and dynamic, occluded targets to better reflect real-world search complexity. The reduced field of view imposed by the HMD likely influenced search behavior, leading to more exaggerated head movements as participants compensated for limited peripheral vision. Similarly, field testing scenarios should consider the effect of helmets or other gear on head movements, as such equipment could substantially alter behavioral patterns compared to those observed in the current laboratory environment.

Our study used a generalized classification model which applied a uniform approach across all participants. While this is useful for identifying overarching patterns in search behavior, it may overlook individual differences in movement strategy, such as varying reliance on head versus eye movement (Fuller, 1992; Stahl, 2001a). An individualized model that accounts for participant-specific movement patterns or preferences could improve predictive accuracy by incorporating eye–head coordination variability and other effector interactions. Refining classification models with personalized metrics would also provide a more detailed perspective on the cognitive mechanisms driving search behavior.

In the present study, we analyzed behavior and eye/head movements across the entire duration of the search process within each trial, without segmenting distinct phases such as the time to first target acquisition, inter-target intervals during the middle of the trial, or the final search segment preceding the last response. This approach was intended to reflect the continuous nature of foraging behavior and to validate that our experimental manipulations influenced task performance. However, we acknowledge that the final segment of a search may involve qualitatively different decisional processes, potentially inflating inter-target times. Future work could examine subcomponents of the search process, such as time to first target, mid-trial search dynamics, or search termination behavior, to better isolate the distinct cognitive processes of each phase.

## Conclusion

This study highlights the benefit of integrating eye and head movement metrics to infer task-induced changes in search behavior. The findings demonstrate that head movements, while often underutilized, offer unique and complementary information to eye metrics. These insights have practical implications for designing adaptive systems for near-real-time monitoring of operator states and informing adaptive systems designed to dynamically adjust their support.

## Supplementary Information

Additional file 1

Additional file 2

Additional file 3

Additional file 4

## Data Availability

The datasets used and/or analyzed during the current study are available from the corresponding author on reasonable request.

## Footnotes

1 RGB color values. Target – (1) 129, 103, 81, (2) 82, 67, 60, (3) 60, 65, 63, (4) 59, 60, 51. Easy distractor – (1) 97, 71, 44, (2) 94, 93, 53, (3) 199, 169, 86, (4) 59, 62, 47. Hard distractor – (1) 113, 86, 68, (2) 78, 86, 56, (3) 88, 80, 68, (4) 77, 71, 63.

2 box plots were created using the boxchart MATLAB (2021b) function. The horizontal line inside each box corresponds to the sample median while the top and bottom edges of the box are the upper and lower quartiles, respectively. Values that are more than 1.5 times the interquartile range (IQR) from the top or bottom are designated as outliers (points beyond whiskers). Each whisker above and below the box represents the maximum value that is not considered an outlier. The top and bottom edges of the notched region correspond to *m* + (1.57⋅*IQR*)/√*n* and *m* − (1.57⋅*IQR*)/√*n*, respectively, where *m* is the median, *IQR* is the interquartile range, and *n* is the number of data points. The green diamond represents the sample mean with gray error bars showing 95% confidence intervals.

3 Top features were extracted and models evaluated without target/distractor feature information and showed the same pattern of results though classification accuracy was generally lower. See Supplementary Materials.
