# Correction: Decoding target discriminability and time pressure using eye and head movement features in a foraging search task

**DOI:** 10.1186/s41235-025-00675-w

**Published:** 2025-09-30

**Authors:** Anthony J. Ries, Chloe Callahan-Flintoft, Anna Madison, Louis Dankovich, Jonathan Touryan

**Affiliations:** 1 Humans in Complex Systems, U.S. Army DEVCOM Army Research Laboratory, 7101 Mulberry Point Rd, Aberdeen Proving Ground, MD 21005 USA; 2 Warfighter Effectiveness Research Center, U.S. Air Force Academy, Colardo Springs, CO 80840 USA

**Correction: Cognitive Research: Principles and Implications (2025) 10:53** 10.1186/s41235-025-00657-y

The original article contained numerous typesetting errors in Table 1 mistakenly carried forward by the production team that handled this article; these errors have since been amended.

