# Supplementary figures and images for: Decoding target discriminability and time pressure using eye and head movement features in a foraging search task
Source: Cogn Res Princ Implic. 2025 Aug 22;10:53. doi: 10.1186/s41235-025-00657-y (PMC12373606; doi:10.1186/s41235-025-00657-y)

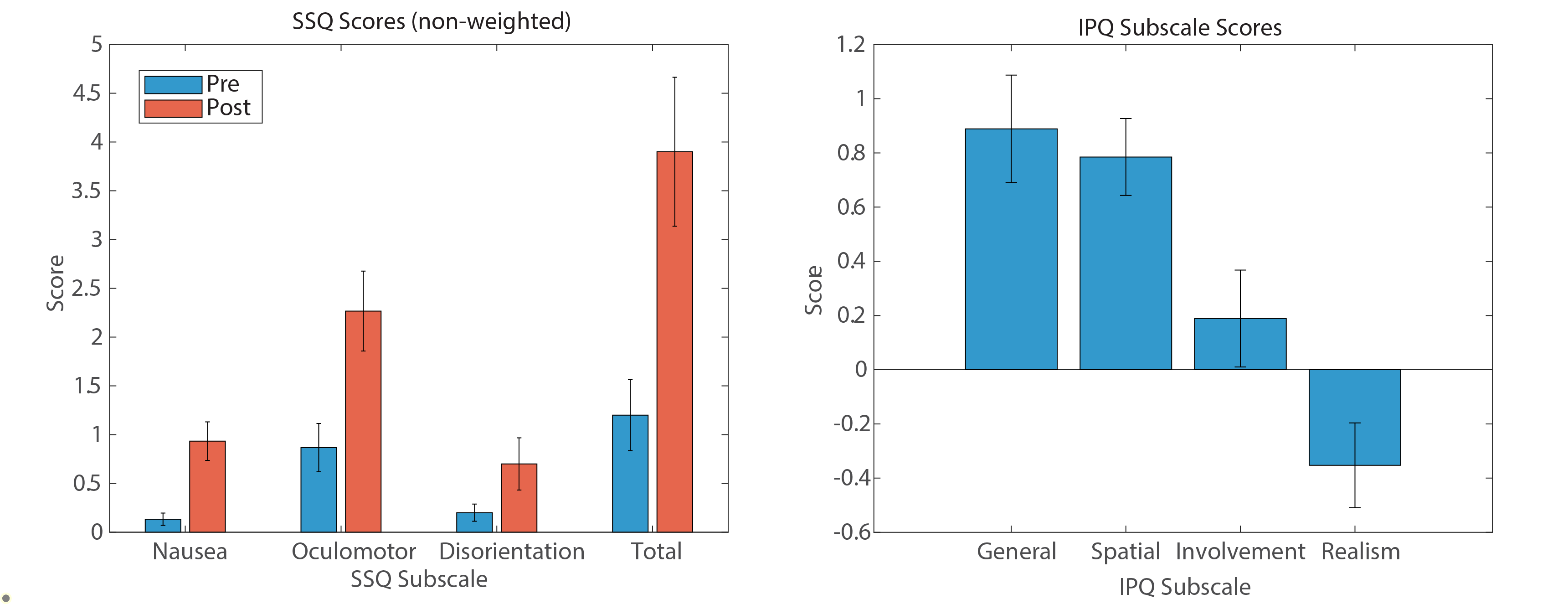

Supplement: Supplementary file 3 — Additional file 3 [file 41235_2025_657_MOESM3_ESM.png]
